# Computer-controlled stimulation for functional magnetic resonance imaging studies of the neonatal olfactory system

**DOI:** 10.1111/apa.12327

**Published:** 2013-08-02

**Authors:** T Arichi, R Gordon-Williams, A Allievi, AM Groves, E Burdet, AD Edwards

**Affiliations:** 1 Centre for the Developing Brain, Division of Imaging Sciences & Biomedical Engineering, Kings College London, St. Thomas’ HospitalLondon, UK; 2 MRC Clinical Sciences Centre, Imperial College London, Hammersmith HospitalLondon, UK; 3 Department of Bioengineering, Imperial College LondonLondon, UK

**Keywords:** fMRI, Infant, Newborn, Olfactory

## Abstract

**Aim** Olfactory sensation is highly functional early in human neonatal life, with studies suggesting that odours can influence behaviour and infant–mother bonding. Due to its good spatial properties, blood oxygen level–dependent (BOLD) contrast functional magnetic resonance imaging (fMRI) has the potential to rapidly advance our understanding of the neural activity which underlies the development of olfactory perception in this key period. We aimed to design an ‘olfactometer’ specifically for use with neonatal subjects for fMRI studies of odour perception.

**Methods** We describe a fully automated and programmable, fMRI compatible system capable of presenting odorant liquids. To prevent contamination of the system and minimize between-subject infective risk, the majority of the olfactometer is constructed from single-use, readily available clinical equipment. The system was used to present the odour of infant formula milk in a validation group of seven neonatal subjects at term equivalent postmenstrual age (median age 40 weeks).

**Results** A safe, reliable and reproducible pattern of stimulation was delivered leading to well-localized positive BOLD functional responses in the piriform cortex, amygdala, thalamus, insular cortex and cerebellum.

**Conclusions** The described system is therefore suitable for detailed studies of the ontology of olfactory sensation and perception during early human brain development.

## Key notes

- Olfaction is important in early life, but the anatomical substrates of the underlying neural activity are poorly understood.
- We describe a fully automated and safe system for fMRI studies of olfaction in neonatal subjects.
- Functional activation can be identified with fMRI in the primary olfactory areas in the neonatal brain.

## Introduction

Through the detection of chemical cues in the environment, the olfactory system is known to play a critical role in guiding survival and social behaviour across the animal kingdom (1). This is particularly evident in mammals immediately following birth, who are directed to the mother’s nipple through olfactory cues, which have been learnt during the in utero period through the odour and taste of the amniotic fluid (2–4). Normal sensory experience during this period appears to be vital for later function, with induced unilateral deprivation at this key juncture shown to markedly affect life-long olfactory function in rats (5). Newborn human infants placed prone on the mother’s chest immediately after delivery, can be shown to preferentially orientate towards an unwashed nipple (as opposed to the washed or rinsed alternative) to initiate breastfeeding (6). In contrast to other sensory systems, reliable behavioural and physiological responses to olfactory stimuli can easily be demonstrated in newborn infants to entirely novel and aversive odorants and have been described in preterm infants as young as 29 weeks postmenstrual age (PMA) (7,8).

However, despite a large volume of behavioural evidence supporting a highly functional neonatal olfactory system, the underlying neuroanatomical substrates in the brain of the human infant are poorly understood (8). In the mature brain, functional neuroimaging studies have found that the olfactory bulbs send primary olfactory information to the piriform cortices on the inferior surface of the brain, with higher level processing occurring in the thalamus, orbito-frontal, cingulate and insular areas (9). Functional near-infrared spectroscopy (fNIRS) can be used to identify changes in cerebral haemoglobin concentration predominately in the orbito-frontal areas in response to olfactory stimuli in neonatal subjects (10,11). However, although multichannel techniques can offer improved localization of brain activity, the optical scattering properties of biological tissue mean that truly accurate source localization can be problematic, particularly for regions deep within the brain. This problem is also prominent in electrophysiological techniques, which may also be affected by induced facial movements leading to significant facial EMG artefacts (12).

Blood oxygen level–dependent (BOLD) contrast functional MRI (fMRI) is commonly used to provide a noninvasive assessment of *in vivo* brain activity in neuroscience studies and increasingly in clinical practice (13). The technique takes advantage of a change in the magnetic properties of haemoglobin when bound to oxygen, allowing it to be used as an endogenous (and therefore entirely noninvasive) contrast agent for functional imaging (13). Through it’s superior spatial specificity and experimental flexibility, the technique has particular potential to rapidly expand our understanding of the development of olfaction, as functional activity can be studied both in the primary olfactory cortex on the inferior surface of the brain and in the discrete areas also known to also be involved in the higher order processing of olfactory stimuli (9,14). While the temporal sensitivity of fMRI is relatively poor in comparison with electrophysiological techniques and NIRS, it is more than sufficient to allow for studies which can either avoid or directly study effects of particular importance in the olfactory system such as habituation and desensitization (14). Although fMRI has previously often given inconsistent results in studies with newborn infants, recent technical advances mean that it is now possible to plan and perform systematic studies with this population (15,16). To allow future studies of the ontogeny of the olfactory system, we aimed to develop a safe and effective fMRI compatible tool for presenting olfactory stimuli at 3.0 Tesla (3T) specifically for newborn infants. We then describe the results of a simple validation experiment in which the described system was used to study the pattern of functional activation induced by a single odorant (formula milk) in a group of infants at term equivalent postmenstrual age (PMA).

## Methods and Materials

The work was approved by the NHS Research Ethics Committee, and written parental consent was obtained prior to all sessions of data acquisition.

### Stimulus system design and implementation

The design of the olfactory stimulus presentation system (‘olfactometer’) was based on similar principles to those previously described in adult fMRI studies, where a carrier gas (usually medical grade breathable air) is directed into an odorant chamber, with the resultant odorized gas delivered directly to the subjects’ nose (17–19). Of importance, the system was designed to be entirely metal free and fMRI compatible (20), and given the fragile nature of the intended neonatal subject group, additional design features were also incorporated to minimize any possible subject discomfort or distress, and limit between-subject infective risk. A list of the parts required for construction of the system can be found in Table S1.

**Figure 1:**
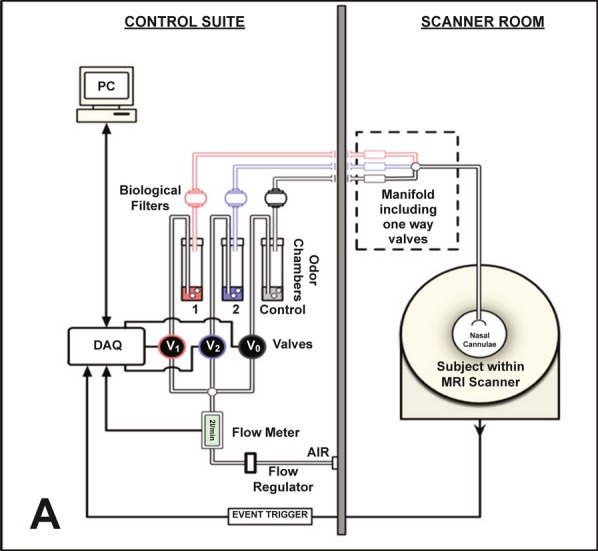
System architecture of the neonatal olfactometer: The olfactometer is composed of three subsystems, with the *airflow preparation* and *odour-sourcing apparatus* situated in the MR scanner console room. Medical grade breathable air is supplied to the system through the standard wall socket, with control and monitoring of air flow possible with a regulator and digital flow meter respectively. Air flow is then directed via the controlled opening of on/off valves into 1 of 3 odour chambers containing an odorant liquid. The vaporized odour is then delivered directly to the subject inside the scanner examination room via nasal cannulae (*delivery apparatus*).

As proposed by Johnson and Sobel ((19)), an olfactometer can be conceptualized into three major subsystems: (i) the airflow preparation apparatus; (ii) the odour-sourcing apparatus; (iii) the delivery apparatus (see Fig. 1). Although custom designed, the described system is constructed entirely from off-the-shelf pneumatic parts (Festo Cooperation, Esslingen am Neckar, Germany); and to minimize infective risk, readily available single-use clinical equipment. Both the airflow preparation and odour-sourcing subsystems are placed in the scanner control room, with precise control of the timing of stimulation achieved via a data acquisition card (DAQ) and custom software (Labview v11.0; National Instruments, Austin, TX, USA) (15). The system airflow (medical grade air) is supplied via the hospital compressed breathing air supply wall socket, with the flow rate into the system (up to 2 L/min) set via a one-way flow control valve. This flow rate was chosen empirically following pilot experiments with adult subjects, and selected at a level at which the odour could be easily detected by the subject without causing any discomfort. As an additional safety feature and to ensure consistency during stimulus delivery, a flow sensor with an LCD display was then integrated into the system to provide an accurate quantification of the air flow entering the system.

**Figure 2:**
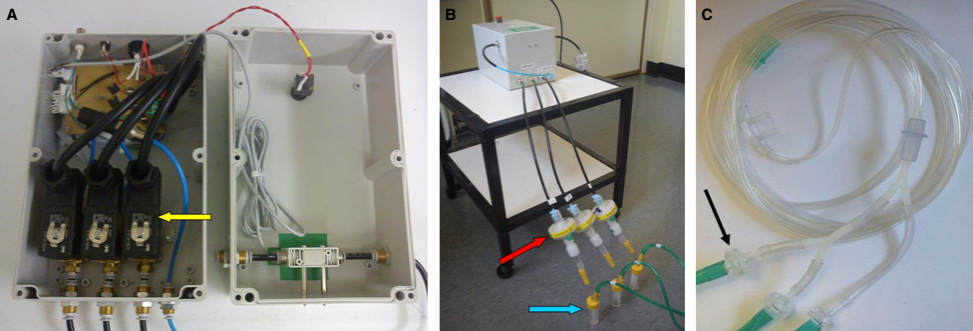
The neonatal olfactometer was developed to be Functional magnetic resonance imaging (fMRI) compatible and to minimize between-subject infective risks: (A): The flow meter, valves (yellow arrow) and data acquisition card (National Instruments, Austin, TX USA) are housed inside a single control box that is situated in the scanner control suite; (B): To minimize infective risk, all components distal to the control box are single-use pieces of readily available clinical equipment such as mucous specimen traps (Pennine Healthcare, Derby, UK) (blue arrow), and antimicrobial respiratory filters are fitted (red arrow). (C): The delivery apparatus contains a manifold containing one-way valves (black arrow) to prevent the mixing of odours. These are then connected to nasal cannulae fitted to the subject prior to data acquisition.

The odour-sourcing subsystem (b) consists of three disposable odour chambers made using single-use mucous specimen traps (Pennine Healthcare, Derby, UK), each containing 3 mL of liquid odorant (see Fig. 2). To prevent possible contamination of the control unit and minimize between-subject infective risk, an anaesthetic grade ventilator circuit breathing filter (Clear-Guard II; Intersurgical Ltd., Wokingham, UK) was fitted to the inlet of each odour chamber. In addition, the use of medical grade air from the hospital wall supply has the important advantage that the airflow has already been filtered for particulate and chemical contamination. Air flow into each of the odour chambers was controlled via separate on/off pneumatic valves, with timed opening and closing achieved via the user interface and DAQ. The system was designed such that the third of the three odour chambers could contain a control odour (sterile water), with the valve to this chamber remaining continuously open between periods of stimulation. During periods of stimulation, the control valve is closed and the selected odorant is presented by simultaneous opening of the appropriate valve. The delivery apparatus (c) is fitted to the subject prior to image acquisition and consists of appropriately sized soft-tip curved nasal cannulae (Flexicare Medical Ltd., Mountain Ash, UK) connected to a manifold containing three one-way valves, which prevent odour mixing (Bio-orb, Reef-One, Norwich UK). Each of these valves is then connected via 6 m lengths of PVC bubble tubing (Flexicare Medical Ltd., Mountain Ash, UK) to the individual odour chambers. To prevent any subject discomfort and maximize patient safety, the system has several means of monitoring and limiting the possible airflow: firstly through the flow control valve; secondly through the stimulus control software which allows only one of the solenoid valves to be opened at a time; thirdly through the stimulus control interface on which a visual feedback graph displays airflow in real time during an experiment; and lastly through two emergency stop switches (one on the device itself and the other on the computer interface), which can immediately close all three of the solenoid valves if required.

### fMRI experimental design

Seven term equivalent neonatal subjects (three male) of median PMA at scan 40 weeks (range from 38 + 4 to 42 + 6 weeks) and median weight at scan 2765 g (range from 2160 to 3420) were recruited from the neonatal intensive care unit and postnatal wards at the Queen Charlotte and Chelsea Hospital, London, UK. Patient information including clinical characteristics and details of their feeding are described in Table.1. The majority of the subjects (6 of the 7 infants) were born prematurely (median gestation at birth 27 + 3 weeks (range 24 + 3 to 37 + 6 weeks). None of the infants required any form of respiratory support at the time of scan, nor had any form of focal brain injury identified on cranial ultrasound scanning. Oral sedation (chloral hydrate 30–50 mg/kg dose) was administered approximately 20 min before the MR imaging session to 6 of the 7 infants studied. All of the infants were assessed clinically prior to the MRI scan by a paediatrician, who was also in attendance throughout the image acquisition period.

**Table 1:** Age and clinical characteristics of the pilot study population

Subject Number	Gestational age at birth (weeks + days)	Postmenstrual age at scan (weeks + days)	Birth weight (and at scan) in grams	Birth occipito-frontal head circumference (and at scan) in millimetres	Sedation
1	37 + 6	38 + 3	3480 (3422)	36 (36)	No
2	27 + 3	40 + 0	1060 (2315)	25.6 (35.6)	Yes
3	29 + 2	39 + 5	1045 (2760)	25.7 (34.3)	Yes
4	24 + 3	41 + 1	535 (2160)	20 (33)	Yes
5	27 + 1	39 + 4	1025 (3080)	26 (35.5)	Yes
6	32 + 2	42 + 6	1340 (3020)	28 (35.5)	Yes
7	25 + 3	42 + 5	850 (2765)	22.8 (34.3)	Yes

In view of the possible effects of stimulus habituation, an event related experimental design was used in which the odour was presented for 6 sec with a 26 sec inter-stimulus period and 10 repetitions. Due to the heterogeneity in the feeding regimes of the subject group, formula milk was used as a single odorant for stimulation (Aptamil; Nutricia Ltd., Wiltshire, UK), thus allowing us to study infants who were both breast and formula milk fed. All of the subjects had been fed at least one hour prior to the imaging session, and did not demonstrate any behavioural signs of hunger at the time of study.

### fMRI image acquisition

MR imaging was performed on a Philips Achieva 3T system (Philips Medical Systems, Best, Netherlands) sited within the neonatal intensive care unit (NICU) using an eight-channel phased-array head coil. The temperature, oxygen saturations and heart rate of the subjects were monitored throughout the period of image acquisition (21). Ear protection was used in all infants in the form of moulded ear-plugs (using a silicone-based putty (President Putty; Coltene Whaledent, Mahwah NJ, USA), and adhesive ear muffs (Minimuffs; Natus Medical Inc., San Carlos, CA, USA). To reduce patient movement during image acquisition, the subjects were also placed in a vacuum-evacuated MRI infant immobilizer (MedVac, Fenton, MI, USA). High-resolution T2-weighted images and 3D MPRAGE T1-weighted images were acquired for all of the subjects, which were then reviewed by a neonatal neuroradiologist [sequence parameters can be found in ref (21)]. BOLD contrast fMRI data were acquired with a single-shot echo-planar imaging (EPI) sequence lasting 6 min and 34 sec with the parameters: (TR) 1500 msec; (TE) 45 msec; (flip angle) 90^°^; [resolution(x*y*z)] 2.5*2.5*3.25 mm; (matrix) 80*80; (slices) 22; (total volumes) 256.

### fMRI data analysis

All imaging data were processed and analysed using tools in the FMRIB software library (FSL, Oxford, UK, www.fmrib.ox.ac.uk/fsl) (22). Standard preprocessing steps were first performed on all fMRI data sets as implemented in FEAT (fMRI Expert Analysis Tool, v5.98) including: motion correction with MCFLIRT (FSL’s intramodal motion correction tool), slice-timing correction, spatial smoothing (FWHM 5 mm), global intensity normalization, temporal filtering (high pass cut-off 50 sec), and denoising of physiological and motion artefacts using MELODIC (Model-free fMRI analysis using probabilistic independent component analysis [PICA, v3.0)] (22). Time-series analysis was then performed using the general linear model (GLM), with the data defined using a convolution of the experimental design with a neonatal-specific hemodynamic response function (HRF) (16). In this approach, the BOLD contrast time series is defined as a convolution of two known variables (the experimental design and the HRF), which is scaled by an unknown parameter estimate (plus the baseline signal and an estimate of the error). For each voxel in the image, a *t*-statistic can therefore be calculated by dividing the parameter estimate by the standard error of the fit, which can therefore be converted to a normalized figure such as a *z*-score. The statistical activation maps derived from this analysis (thresholded at *z* = 2.3 with a corrected cluster significance level of p < 0.05) were then registered to the individual subject’s high-resolution T2-weighted image for visualization, and then to a custom standard template brain using FMRIB’s linear image registration tool (FLIRT) v5.5 (15,22). Higher level group analysis was then performed in standard template space using a nonparametric one-sample *t*-test using permutation methods implemented in Randomize (v2.1) and threshold-free cluster enhancement (TFCE) (23). A false discovery rate (FDR) calculation was used to correct for multiple comparisons with a threshold of p < 0.05.

## Results

Blood oxygen level dependent contrast functional data were successfully collected in all seven of the studied infants, with no adverse events occurring during the imaging session or in relation to the olfactory stimulus. Four of the infants were exclusively breastfed at the time of data acquisition, while the three other infants were fed with both formula milk and breastmilk. Although a degree of head motion was noted in all of the infants during the fMRI data acquisition, it was not found to be increased or correlated with the timing of stimulus presentation. No changes in the recorded physiological parameters were observed during periods of olfactory stimulation.

**Figure 3:**
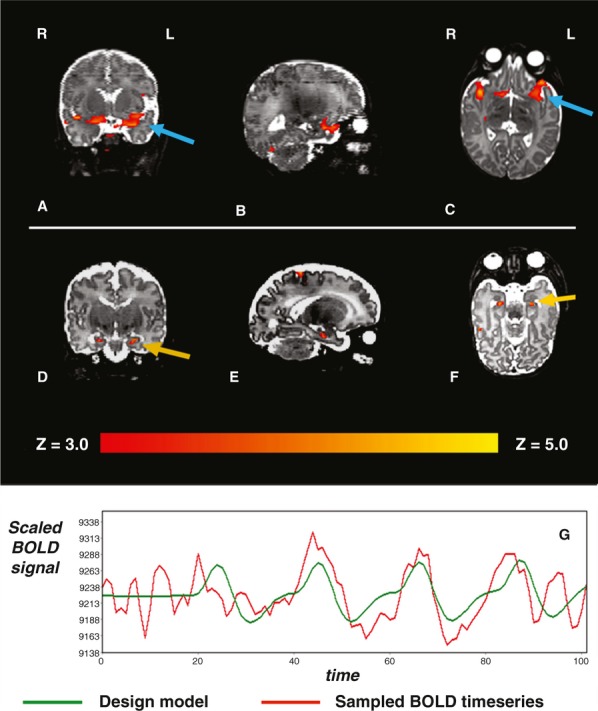
Example olfactory responses to the odour of formula milk in two ex-preterm infants at term equivalent postmenstrual age (PMA). (A,B,C): Coronal (A), sagittal (B) and axial (C) T2-weighted images with an overlaid thresholded statistical map (corrected cluster significance of p < 0.05) showing well-localized functional responses in the piriform cortices bilaterally in an infant at 42 + 6 weeks PMA (blue arrows). (D,E,F): Well localized responses were also identified in the amygdalae as shown in an infant at 39 + 6 weeks PMA (yellow arrows). (G): In the example time series (averaged over the clusters of activation), the sampled Blood oxygen level dependent (BOLD) signal (red) can be seen to closely fit the predicted response as modelled by the pattern of stimulation and an age-appropriate Hemodynamic response function (HRF) model (green).

In the single-subject analysis, well localized increases in BOLD signal in keeping with positive functional activity could be identified in majority (5/7) of subjects in the area of the piriform cortices (4/7 bilateral) (see Table. 2). A diffuse pattern of functional activity was seen in all seven subjects in the cerebellum, the insular cortex (5/7), and less commonly in the orbito-frontal regions (3/7). No consistent patterns of negative BOLD contrast change were seen in any of the subjects (see Fig. 3).

**Table 2:** Spatial localization of identified responses in the study population (“tick” denotes a positive response identified in the subject)

		Pattern of activation				
Subject number	Feeding regime	Piriform cortex	Thalamus	Cerebellum	Orbito-frontal areas	Insula
1	Breastfeeding exclusively	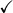	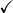	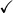	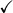	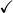
2	Breast and formula feeding	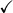	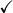	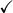	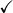	–
3	Breast and formula feeding	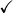	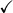	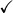	–	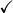
4	Breast and formula feeding	–	–	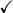	–	–
5	Breastfeeding exclusively	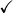	–	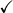	–	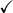
6	Breastfeeding exclusively	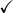	–	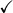	–	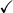
7	Breastfeeding exclusively	–	–	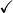	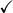	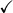

**Figure 4:**
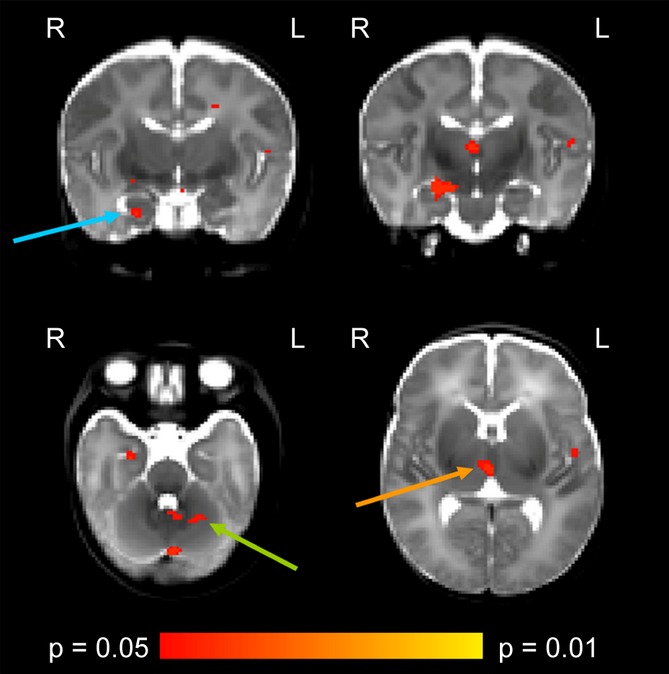
The average response to the odour of formula milk in seven infants at term equivalent postmenstrual age. The results of a one-sample nonparametric *t*-test (with a false discovery rate correction for multiple comparisons at threshold p < 0.05) have been overlaid on a custom T2-weighted template image in the coronal (A,B) and axial (C,D) planes. Clusters of activation are seen in the right piriform cortex and amygdala (blue arrows), the right thalamus (orange arrow), the left insular area (white arrow) and cerebellum (green arrow).

The group analysis identified a widely dispersed but discrete pattern of activation, similar to that described in olfactory fMRI studies in adult subjects (14). Well-localized clusters of activation were seen in the right piriform cortex, the right thalamus, the left insular area and cerebellum (see Fig. 4). The activity in the orbito-frontal regions seen on the lower-level analyses in a subgroup of subjects did not reach significance in the group analysis.

## Discussion

We describe the design and implementation of a custom device that allows fMRI studies of olfaction in newborn infant subjects. In addition to incorporating features that make the system suitable and safe for use with the specific subject group, it is fully automated and programmable and is capable of providing a reliable and reproducible pattern of stimulation. In seven infants at term equivalent PMA, the system induced a well-localized pattern of positive BOLD functional activity in many of the areas that are associated with primary olfaction in the mature adult brain (14).

While the conceptual framework of an automated ‘olfactometer’ has long been known and utilized in neuroscience (17), it is only recently that they have been adapted for use in the environment of the MRI scanner (18,19). Previous studies of olfactory function specifically in neonatal subjects have employed manual approaches such as soaked gauze (10,11); odorant applied to the mother’s chest (6); or manually controlled airflow (24). The described new system combines fMRI compatibility, a reproducible pattern of stimulation, and experimental flexibility in that functional responses to theoretically any liquid, dissolved or suspended odorant can be studied and directly compared. Furthermore, as both the experimental paradigm and airflow rate can be accurately selected by the investigator, salient olfactory effects such as habituation and desensitization can be accounted for or studied (14). Particular care was taken during the design and implementation of the olfactometer, with the use of single-use readily available clinical equipment where possible. While this approach was taken so as to importantly limit potential between-subject infective risk and costs, it does have the potential drawback that the PVC tubing used has its own detectable odour and that odorants can adhere to the inner surface of the tubing (25).

In the validation study, the odour of formula milk elicited a well-localized pattern of functional brain activity in the primary olfactory areas, and specifically in the piriform cortex. Due to the small subject group and differences in exposure prior to image acquisition, the results cannot be regarded as representative of the specific response to formula milk, and therefore, the implications of the more distributed activity in the associative areas (insula, thalamus and cerebellum) are unclear. In the mature brain, the olfactory system is known to contain a complex associative network which exhibits rapid adaptive plasticity and contributes to a rich perception of odour including associative memories and emotion (1). While the level of this complexity is unknown in the newborn brain, animal studies have suggested that exposure to a variety of odours shortly after birth correlates with marked changes in the cellular organization of the piriform cortices and that deprivation at this key juncture (but not later in life) can lead to significant long-term effects on later function (2,5). Behavioural studies have suggested that the olfactory system in newborn human infants is indeed highly functional and is capable of distinguishing between familiar, pleasant and aversive smells (8). The odour of breastmilk has also been shown to have clinically relevant effects such as decreasing behavioural measures of pain and increasing the objective rate of non-nutritive sucking (24,26). Through the pruning and strengthening of association fibres, the olfactory cortex is thought to act as a highly complex pattern recognition device, capable of a variety of processes including odour discrimination, perceptual stability and historical/memorial smell association (1). Olfactory sensory neurons are continuously replenished throughout life, and therefore, the system is characterized by a marked capacity for relatively rapid sensory-driven plasticity (27). Human newborn infants can undergo classical conditioning to olfactory stimuli, and the described system therefore offers the exciting possibility that the neural correlates which underlie early associative learning can be studied (28,29).

Induced light sedation with chloral hydrate is often used during MRI scanning of young infants to reduce subject distress and motion artefact. Positive and well-localized BOLD responses can be identified in sleeping infants, and chloral hydrate has not been found to alter either the induced fMRI responses, or underlying baseline cerebral blood flow (15,16). In addition, it has been shown that the brain is capable of learning new associations between olfactory and auditory stimuli even during deep sleep and to preserve them during subsequent awake testing (30). However, deep sedation with anaesthetic agents may interfere with the ability of the olfactory system to discriminate between odours, and future studies may therefore need to be carried out with awake infants to demonstrate these specific effects.

## Conclusions

We have designed, constructed and implemented a fully automated and programmable olfactometer specifically for fMRI studies of neonatal subjects. The described system is safe, has been designed to minimize infective risks and produces a reproducible but flexible pattern of olfactory stimulation. The system was found to induce a well-localized pattern of positive BOLD functional activity in the primary olfactory areas of seven neonatal subjects at term equivalent PMA. These data suggest that fMRI now offers the opportunity to investigate the ontogeny of olfaction in the human newborn with millimetre-scale precision, and to address specific hypotheses concerning the development of mature olfactory responses.

## Abbreviations

BOLD: Blood oxygen level dependent
DAQ: Data acquisition card
EPI: Echo-planar Imaging
FDR: False discover rate
FWHM: Full-width half-maximum
GLM: General linear model
fMRI: Functional magnetic resonance imaging
fNIRS: Functional near-infrared spectroscopy
HRF: Hemodynamic response function
LCD: Liquid crystal display
MPRAGE: Magnetization-prepared rapid acquisition gradient echo
NHS: National Health System (UK)
PMA: Postmenstrual age
PVC: Polyvinyl chloride
3T: 3.0 Tesla
TE: Echo time
TR: Repetition time

## SUPPORTING INFORMATION

Table S1 List of parts required for production of the described olfactometer system, with the fittings required for a single experiment.
