# Supplementary material for: Computer-controlled stimulation for functional magnetic resonance imaging studies of the neonatal olfactory system
Source: Acta Paediatr. 2013 Aug 2;102(9):868–75. doi: 10.1111/apa.12327 (PMC3795441; doi:10.1111/apa.12327)
Supplement: Table S1 — List of parts required for production of the described olfactometer system, with the fittings required for a single experiment. [file apa0102-0868-sd1.doc]

| **Part** | **Supplier** | **Amount required** | **Cost** |
| --- | --- | --- | --- |
| Flow regulator ARM55A-06-A | RS components | 1 | £28.16 |
| Solenoid Valve Vx2120-02F-5D1-B | SMC pneumatics | 3 | £53.94 |
| Data Acquisition Card USB-6009 | National Instruments | 1 | £179.00 |
| Air flow switch with display PFM710-C6-E | RS components | 1 | £167.60 |
| Multiple airflow distributor QS, 3 outlet | Festo Corporation | 1 | £3.50 |
| PUN plastic tubing 3mm diameter | Festo Corporation | 1.5m | £2.19 |
| Wht one touch straight adaptor KQ2H06-02S | RS components | 3 | £5.28 |
| BNC bulkhead connector | RS components | 1 | £3.93 |
| Clearguard II air filter | Intersurgical UK | 3 | £3.30 |
| Bubble oxygen tubing | Flexicare Medical | 3*6m | £1.08 |
| Neonatal nasal cannulae | Flexicare Medical | 1 | £0.89 |
| Mucous trap | Pennine Healthcare | 3 | £4.80 |
| One way valve | Reef-One | 3 | £9.00 |
| **Total** |  |  | **£452.67** |

**Supplementary table:** *List of parts required for production of the described olfactometer system, with the fittings required for a single experiment. Parts in italics are intended for single use only, and are discarded after each subject. Please note, the total cost described does not include the PC and Labview software (National Instruments, Austin TX, USA) required to control the olfactometer system.*
